# Satellite Cells Exhibit Decreased Numbers and Impaired Functions on Single Myofibers Isolated from Vitamin B6-Deficient Mice

**DOI:** 10.3390/nu13124531

**Published:** 2021-12-17

**Authors:** Takumi Komaru, Noriyuki Yanaka, Thanutchaporn Kumrungsee

**Affiliations:** Graduate School of Integrated Sciences for Life, Hiroshima University, 1-4-4 Kagamiyama, Higashi, Hiroshima 739-8528, Japan; m200189@hiroshima-u.ac.jp (T.K.); yanaka@hiroshima-u.ac.jp (N.Y.)

**Keywords:** sarcopenia, vitamin B6, skeletal muscle, single myofibers, satellite cells, proliferation, self-renewal, amino acids, carnosine

## Abstract

Emerging research in human studies suggests an association among vitamin B6, sarcopenia, and muscle strength. However, very little is known regarding its potential role at the cellular level, especially in muscle satellite cells. Therefore, to determine whether vitamin B6 affects the satellite cells, we isolated single myofibers from muscles of vitamin B6-deficient and vitamin B6-supplemented mice. Subsequently, we subjected them to single myofiber culture and observed the number and function of the satellite cells, which remained in their niche on the myofibers. Prior to culture, the vitamin B6-deficient myofibers exhibited a significantly lower number of quiescent satellite cells, as compared to that in the vitamin B6-supplemented myofibers, thereby suggesting that vitamin B6 deficiency induces a decline in the quiescent satellite cell pool in mouse muscles. After 48 and 72 h of culture, the number of proliferating satellite cells per cluster was similar between the vitamin B6-deficient and -supplemented myofibers, but their numbers decreased significantly after culturing the myofibers in vitamin B6-free medium. After 72 h of culture, the number of self-renewing satellite cells per cluster was significantly lower in the vitamin B6-deficient myofibers, and the vitamin B6-free medium further decreased this number. In conclusion, vitamin B6 deficiency appears to reduce the number of quiescent satellite cells and suppress the proliferation and self-renewal of satellite cells during myogenesis.

## 1. Introduction

Sarcopenia is a progressive muscle disease that is characterized by a decline in the skeletal muscle mass as well as function (strength and physical performance) with advancing age. Risk factors for sarcopenia include body composition, pharmacological therapy, lifestyle habits, intrinsic factors, and diet [1]. Loss of muscle mass, an important pathophysiological symptom of sarcopenia, is mainly attributed to an imbalance between muscle synthesis and degradation, which appears to increase due to aging [2]. Predominant changes that occur at the cellular level of sarcopenia-affected muscles include a decrease in the size as well as number of myofibers and a reduction in the number and function of satellite cells. In fact, these satellite cells are muscle stem cells that play an important role in muscle regeneration, which is essential in maintaining muscle mass during aging [1,2].

Currently, there are no medical treatments for sarcopenia. In fact, the main recommendations for sarcopenia management are exercise and nutritional interventions [3,4]. Even though the role of nutrition in the prevention of sarcopenia forms a key field of research [5], extensive studies are still necessary to establish this fact. With respect to the role of nutrients in improving muscle mass and strength, the majority of the studies to date have extensively reported on the functions of proteins and amino acids, followed by vitamin D and omega 3 fatty acids [3,5,6]. Interestingly, among the 13 vitamins in humans, only vitamin D has been extensively studied at both physiological and muscle cell levels. However, emerging research over the last 5 years suggests that other nutrients and vitamins may be potentially involved in sarcopenia [4,7,8,9,10,11,12,13]. In fact, human studies in both European countries and Japan have demonstrated that low intake as well as low levels of vitamin B6 coexist with sarcopenia [7,8,9,10,11]. Although these reports provide crucial evidence for the association of vitamin B6 with sarcopenia, the evidence has largely been obtained from observational studies.

Hence, in this study, we examined the cellular-level effects of vitamin B6 on the number and function of satellite cells. We applied a single myofiber technique that allowed us to observe the number and function of the satellite cells, while they remained in their niche on the myofibers, thereby ensuring that the conditions resemble the normal physiological state. To determine whether vitamin B6 deficiency can affect the number and function (self-renewal, proliferation, and differentiation) of the satellite cells, we isolated single myofibers from the skeletal muscles of vitamin B6-deficient and -supplemented mice. Subsequently, we cultured the myofibers for 0, 24, 48, and 72 h and ultimately observed the number and function of the satellite cells on them. Since the main function of vitamin B6 is to be a cofactor for enzymes required in amino acid metabolism [14,15], its deficiency is suspected to affect amino acid and protein synthesis in sarcopenia [7,8,11]. Therefore, we further examined whether vitamin B6 deficiency can induce changes in the levels of amino acids and major muscle protective peptides, namely carnosine and anserine, in skeletal muscles. We believe that this study will provide new insights into the role of vitamin B6 with respect to satellite cells and sarcopenia.

## 2. Materials and Methods

### 2.1. Animals and Diet

Male CD-1 (ICR) mice (4-w-old, Charles River Japan, Hino, Japan) were maintained in accordance with the Guide for the Care and Use of Laboratory Animals established by Hiroshima University and approved by the Ethics Committee of the University (Ethical Approval No. C18-15-5). The mice were housed in metal cages in a temperature-controlled room (24 ± 1 °C) under a 12 h light/dark cycle (lights on, 08:00–20:00 h). They had free access to food and drinking water and were acclimatized to a non-purified commercial rodent diet (MF, Oriental Yeast, Tokyo, Japan) for 7 d before experimental treatments. Thereafter, 20 mice were randomly divided into 2 groups (*n* = 10/group), wherein they received a basal diet mixed with 1 mg (marginal deficient) or 35 mg (supplemented) pyridoxine (PN) HCl/kg diet for 8 w. The basal diet included the following components (g/kg diet): α-cornstarch, 402; casein, 200; sucrose, 200; corn oil, 100; cellulose, 50; AIN-93G mineral mixture, 35; AIN-93 vitamin mixture (PN free), 10; and L-cystine, 3, as described previously [16,17]. Usually, 7 mg PN HCl/kg diet is the recommended level of dietary vitamin B6 [16]. Additionally, 1 mg PN HCl/kg diet is the marginal deficient level, i.e., the minimum level required to prevent vitamin B6 deficiency-induced growth depression [18], and 35 mg PN HCl/kg diet is the maximum supplemented level reported to have no toxicity [17,19]. To compare the effects of low dose and high dose dietary supplementation of vitamin B6, similar to our previous study [17], on satellite cells, we selected 1 mg PN HCl/kg diet (the low or deficient level) and 35 mg PN HCl/kg diet (the high or supplemented level) in the present study. The PN HCl was obtained from Nacalai Tesque (Kyoto, Japan). After completion of the experiment, all the mice were sacrificed under isoflurane anesthesia (between 13:00 and 15:00 h), 6 h after food removal. Blood was collected from the abdominal vein into tubes containing heparin as an anticoagulant on ice. Subsequently, plasma was obtained by centrifugation at 3500 rpm for 10 min and stored at −80 °C. Gastrocnemius (GAS) muscles were harvested immediately, weighed, snap-frozen in liquid nitrogen, and stored at −80 °C, until further analysis. Additionally, the extensor digitorum longus (EDL) muscles were harvested and subjected to single myofiber isolation.

### 2.2. Single Myofiber Isolation and Culture

**Figure 1:**
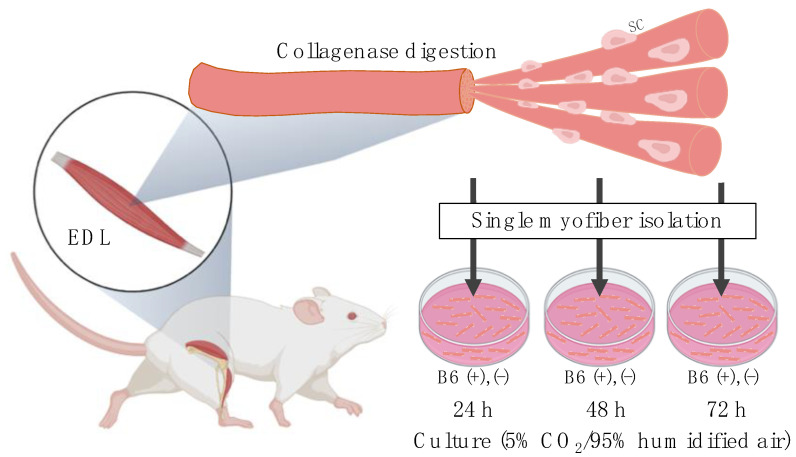
Experimental procedure of single myofiber culture for assessing satellite cell number and function while the cells remain in their niche on single myofibers. EDL, extensor digitorum longus; SC, satellite cells; B6, vitamin B6 (PN HCl). Created with BioRender.com (accessed on 12 December 2021).

Single myofiber isolation and culture was performed, according to a previously described protocol [20], with some modifications. As shown in Figure 1, the EDL muscles were harvested and digested using 0.2% *w*/*v* collagenase type I (Invitrogen, Carlsbad, CA, USA) by incubating at 37 °C for 2 h. Single myofibers were triturated from the digested muscle bundles and transferred to 10% horse serum-coated cell culture dishes using a glass Pasteur pipette. Thereafter, these isolated single myofibers were either fixed with 4% paraformaldehyde (PFA) immediately (0 h) or cultured at 37 °C for 24, 48, or 72 h, under controlled conditions of 5% CO_2_/95% humidified air. The culture medium consisted of high-glucose (4.5 g/L) vitamin-free Dulbecco’s modified Eagle’s medium (DMEM; Research Institute for the Functional Peptides, Yamagata, Japan), 10% horse serum (Sigma), 0.5% chick embryo extract (US Biological Life Sciences, Salem, MA), 1% penicillin-streptomycin, and certain vitamins (mg/L at a final concentration), namely calcium pantothenate (4.0); choline chloride (4.0); folic acid (4.0); inositol (7.2); niacinamide (4.0); riboflavin (0.4); thiamine HCl (4.0); and PN HCl (4.0). To maintain the vitamin B6-deficient myofibers under vitamin B6-deficient conditions throughout the culture period, these myofibers were separately cultured without adding the PN HCl in the medium (hereafter referred to as 1 mg (−)). After 24, 48, or 72 h of culture, the single myofibers were fixed with 4% PFA and subjected to immunofluorescence staining to determine the number and function (self-renewal, proliferation, and differentiation) of the satellite cells remaining on the myofibers.

### 2.3. Immunofluorescence Staining

The PFA-fixed single myofibers were washed using phosphate buffered saline (PBS) and incubated in 1% *v*/*v* Triton X-100 for 30 min, followed by 30 min in blocking buffer (1.5% *v*/*v* normal goat serum and 1.5% normal horse serum) at room temperature to prevent non-specific binding. Subsequently, the myofibers were incubated with primary antibodies against paired homeobox 7 (Pax7; 1:3; monoclonal mouse anti-Pax7 IgG; Developmental Studies Hybridoma Bank (DSHB), Iowa City, IA, USA) or co-stained for both Pax7 and myogenic factor D (MyoD; 1: 100; polyclonal rabbit anti-MyoD IgG; Santa Cruz Biotechnology M-318) for 18 h (overnight) at 4 °C. The mouse primary antibody was visualized using FluoroLinkCy3-labeled goat anti–mouse IgG (1:1000; Amersham Biosciences PA43002), while the rabbit primary antibody was visualized using fluorescein (FITC)-AffiniPure goat anti-rabbit IgG (1:500; Jackson Immuno Research 111-095-003). The entire fluorescent antibody labeling was performed at room temperature for 45 min. All antibodies were diluted in the blocking buffer. Following PBS-T wash, the nuclei were counterstained with 4′,6-diamidino-2-phenylindole (DAPI), and the myofibers were mounted using a fluorescent mounting medium. All images were acquired using an Olympus BX53 microscope (Olympus, Tokyo, Japan). The number of satellite cells was determined manually by blind counting under the microscope.

### 2.4. Pyridoxal 5′-Phosphate (PLP) Analysis

The PLP concentrations were measured, according to methods described previously [17]. Initially, the GAS muscles and plasma were extracted using 1 N perchloric acid. Thereafter, the PLP (Nacalai Tesque) was converted to pyridoxic acid 5-phosphate by incubation with 0.1 mol/L KCN at 50 °C for 3 h. Subsequently, its levels were measured by high-performance liquid chromatography (HPLC) using a fluorometric detector with an excitation wavelength (Ex) of 305 nm and an emission wavelength (Em) of 390 nm. A Cosmosil 5C18-MS-II column (4.6 × 150 mm; Nacalai Tesque) with an isocratic elution of 1% (*v*/*v*) acetonitrile-0.1 mol/L NaClO_4_-0.1 mol/L phosphate buffer (pH 3.5) was used.

### 2.5. Amino Acid and Imidazole Dipeptide Analysis

To measure the concentration of amino acids in the skeletal muscle tissues, the GAS muscle tissue was homogenized in methanol. After centrifugation, the supernatants were concentrated by evaporation to dryness and resuspended in the first buffer of an amino acid analyzer. Subsequently, the amino acid concentrations were quantified using an amino acid analyzer (JLC-500; JEOL, Tokyo, Japan), according to the manufacturer’s instructions. A mixture of standard amino acid solutions (type AN2 and type B; FUJIFILM Wako Pure Chemical Corporation, Osaka, Japan), L-tryptophan (Nacalai Tesque), L-glutamine (Nacalai Tesque), and L-asparagine (Nacalai Tesque) was used as the standard.

To measure the concentration of the imidazole peptides, namely anserine and carnosine, the GAS muscle tissue was homogenized in methanol containing an internal standard (20 μM methionine sulfone), as previously described [21]. Thereafter, the supernatants were concentrated by evaporation to dryness and resuspended in methanol before further analysis. Anserine and carnosine (Wako Pure Chemical Industries, Osaka, Japan) were detected by ultra-performance liquid chromatography tandem mass spectrometry (UPLC-MS/MS; Waters, Milford, MA, USA), according to a previously described protocol [22] with some modifications. Liquid chromatography was performed at 30 °C using an Acquity UPLC BEH C18 (1.7 μm, 2.1 × 50 mm) column (Waters) and a gradient system with the mobile phase consisting of buffer A (5 mM perfluoroheptanoic acid (PFHpA; Sigma-Aldrich, Louis, MO) in Milli-Q water) and buffer B (5 mM PFHpA in methanol at a flow rate of 400 μL/min). The gradient program began with an initial condition of 95% A and 5% B. Within 10 min, the linear gradient of B was 40 to 50%, and in the next 0.5 min, it became 50 to 100%, with a holding period of 1 min. The gradient was finally reinstated to the initial conditions in 0.5 min with equilibration for 5 min before the next injection. The run-to-run time was 17.5 min, and the injected volume was 5 μL. Mass spectrometric analysis was performed using multiple reaction monitoring (MRM) in the ESI-positive mode. The dissolution temperature was 400 °C, and the source temperature was 120 °C. The capillary voltage, cone voltage, and MRM for anserine and carnosine, respectively, were set as capillary voltage (kV): 3 and 3; cone voltage (V): 15 and 10; and MRM (*m*/*z*): 241 > 109.2 and 227.1 > 110.1. Nitrogen gas was used for both the dissolution and cone gas flows. The MRM and daughter-ion scans were performed using argon as the collision gas. β-Alanine (Nacalai Tesque) was tracked by an *o*-phthalaldehyde (OPA)-based HPLC method, as described previously [21]. A Cosmosil 5C18-MS-II column (4.6 × 150 mm; Nacalai Tesque) with an isocratic elution of 0.1 mol/L of a mixture containing sodium citrate (pH 3.5)/acetonitrile/methanol (60:30:10 (*v*/*v*)) was used. A fluorescence detector set at an Ex of 350 nm and Em of 440 nm was used to monitor the compound.

### 2.6. Muscle Cell (C2C12) Culture

The C2C12 cell line (3.0 × 10^5^ cells/well of a 6-well plate) was cultured in a proliferation medium, with or without vitamin B6, in an incubator under controlled conditions of 37 °C and 5% CO_2_/95% humidified air. The proliferation medium was composed of high-glucose vitamin-free DMEM (Research Institute for the Functional Peptides), 10% fetal bovine serum, 1% streptomycin, and vitamins (mg/L at a final concentration), including calcium pantothenate (4.0); choline chloride (4.0); folic acid (4.0); inositol (7.2); niacinamide (4.0); riboflavin (0.4); thiamine HCl (4.0); and PN HCl (4.0). For the vitamin B6-deficient medium, PN HCl was not added. At 80% confluency (after 2 d), the cells were transferred to a differentiation medium, with or without vitamin B6. The differentiation medium was composed of high-glucose vitamin-free DMEM, 2% horse serum, 1% streptomycin, and the abovementioned vitamins. For the vitamin B6-deficient medium, PN HCl was not added. After 4 d of the differentiation, MilliQ water (0.5 mL/well) was added, and the C2C12 cells were collected by scraping for further analysis. Subsequently, the cell extracts were centrifuged at 12,000× *g* for 10 min at 4 °C. The supernatants were analyzed for the presence of anserine, carnosine, and β-alanine, as described above. The protein concentration in each supernatant was determined with a Bio-Rad DC Protein Assay Kit (Hercules, CA, USA) using bovine serum albumin as the standard.

### 2.7. Statistical Analysis

Data are expressed as mean ± standard deviation (SD). Statistical analysis was performed by unpaired *t* test using GraphPad Prism8 (GraphPad Software, CA, USA). For all the tests, statistical significance was set at *p* < 0.05.

## 3. Results

### 3.1. Food Intake, Body Weight, Muscle Mass, and PLP Levels

Compared to the vitamin B6-supplemented (35 mg PN HCl/kg diet) group, the vitamin B6-deficient (1 mg PN HCl/kg diet) group does not exhibit any significant differences with respect to food intake, final body weight, or skeletal muscle weight (*p* > 0.05, Table 1). The average muscle fiber area from the vitamin B6-deficient group is slightly smaller, but not significantly, than that from the vitamin B6-supplement group (*p* = 0.0593, Supplementary Figure S1). On the contrary, the PLP levels in the plasma as well as muscle tissues are significantly lower in the vitamin B6-deficient group as compared to that in the vitamin B6-supplemented group (plasma: 3.03 ± 1.09 (35 mg) vs. 0.98 ± 0.21 (1 mg) nmol/mL, *p* = 0.003; GAS muscles: 9.9 ± 1.6 (35 mg) vs. 4.5 ± 0.6 (1 mg) nmol/g, *p* < 0.0001; Table 1).

### 3.2. Effects of Vitamin B6 Deficiency on Number and Function of Satellite Cells

#### 3.2.1. Number of Satellite Cells at the Quiescent State and Their Viability after Being Activated

**Figure 2:**
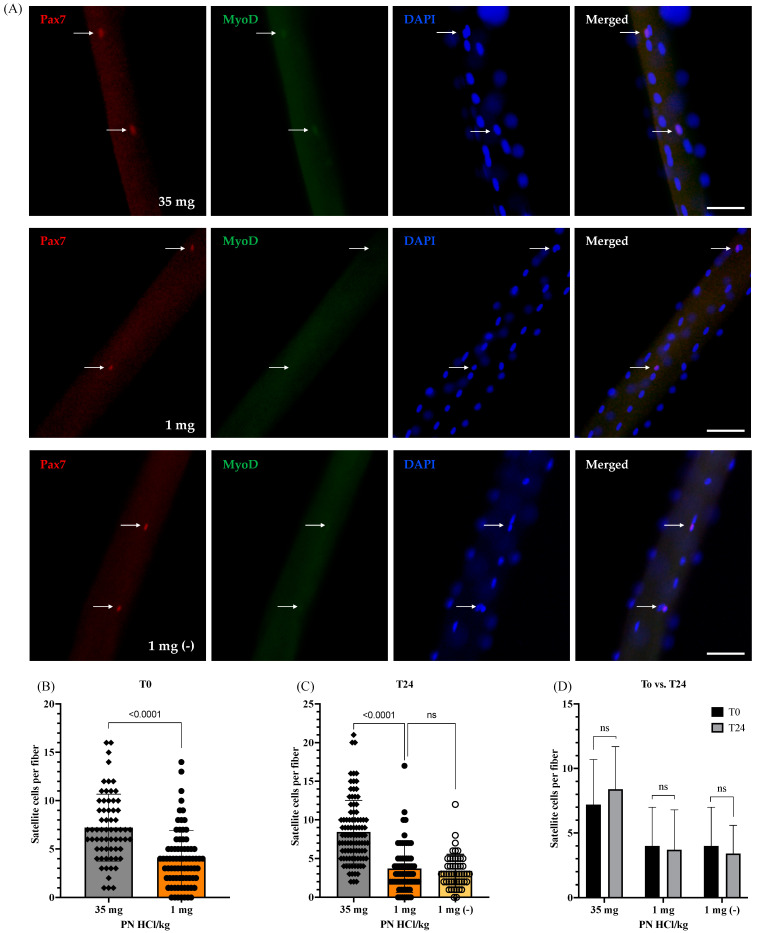
Effects of vitamin B6 deficiency on satellite cell numbers at the quiescent state (0 h) and viability after being activated for 24 h. (**A**) Representative images demonstrating satellite cells stained with Pax7 (arrows) and MyoD on a single fiber at 24 h of culture (scale bar = 50 μm). (**B**) Quantified data showed the number of quiescent satellite cells on myofibers without culturing (0 h of culture) (4 mice were used per group, ≥15 myofibers per mouse; *n* = 65 myofibers (35 mg) and *n* = 77 (1 mg)). (**C**) Quantified data showed the number of activated satellite cells on myofibers following 24 h of culture (3–4 mice were used per group, ≥15 myofibers per mouse; *n* = 95 myofibers (35 mg), *n* = 65 myofibers (1 mg), and *n* = 50 myofibers (1 mg (−))). (**D**) Comparison of the number of satellite cells per myofibers between 0 h (b) and 24 h (c) of culture. 1 mg (−) indicates the vitamin B6-deficient myofibers cultured in the vitamin B6-free medium. 35 mg PN HCl/ kg and 1 mg PN HCl/kg represent the vitamin B6-supplemented group and the vitamin B6-deficient group, respectively. Values represent the means ± SD. *p* value < 0.05 was considered statistically significant (unpaired *t*-test). ns = no significance.

To examine the effects of vitamin B6 deficiency on the number of satellite cells, single myofibers, freshly isolated from the EDL muscles of vitamin B6-deficient and -supplemented mice, were immediately fixed and subjected to immunofluorescence staining for Pax7, a specific satellite cell marker. Figure 2A shows the specific binding of the Pax7 antibody to the Pax7 molecule in the satellite cells on single myofibers. Moreover, prior to culturing (0 h or T0), the number of satellite cells in the quiescent and near physiological state present on the vitamin B6-deficient mouse myofibers is lower than that in the vitamin B6-supplemented mouse myofibers (7.2 ± 3.5 (35 mg) vs. 4.0 ± 3.0 (1 mg) satellite cells/myofiber, *p* < 0.0001), as shown in Figure 2. This suggests that the vitamin B6 deficiency possibly impairs satellite cell synthesis or induces a decline in the quiescent satellite cell population in mouse muscles.

To determine whether vitamin B6 deficiency affects the ability of satellite cells to be activated, the single myofibers isolated from EDL muscles of both vitamin B6-deficient and -supplemented mice were incubated in the culture medium containing normal vitamin B6 levels for 24 h. Additionally, to maintain the vitamin B6-deficient myofibers under vitamin B6-deficient conditions throughout the ex vivo culture, they were simultaneously cultured in a medium lacking vitamin B6 (1 mg (−)). Incidentally, after 24 h of culture (T24), the satellite cells are in the activated state. We observed that the number of activated satellite cells on the vitamin B6-deficient mouse myofibers is significantly lower than that in the vitamin B6-supplemented mouse myofibers at T24 (8.1 ± 4.1 (35 mg) vs. 3.7 ± 3.1 (1 mg) satellite cells/myofiber, *p* < 0.0001); however, there are no significant differences between the number of activated satellite cells in the vitamin B6-deficient mouse myofibers and the ones cultured in the vitamin B6-deficient medium (1 mg (−); 3.4 ± 2.2 satellite cells/myofiber), as shown in Figure 2C. Furthermore, we did not observe any significant difference in the number of satellite cells between the quiescent state (T0) and the activated state (T24) on the vitamin B6-deficient and -supplemented mouse myofibers as well as on the vitamin B6-deficient mouse myofibers cultured in the vitamin B6-deficient medium, as shown in Figure 2D.

#### 3.2.2. Satellite Cell Function: Proliferation, Self-Renewal, and Differentiation

**Figure 3:**
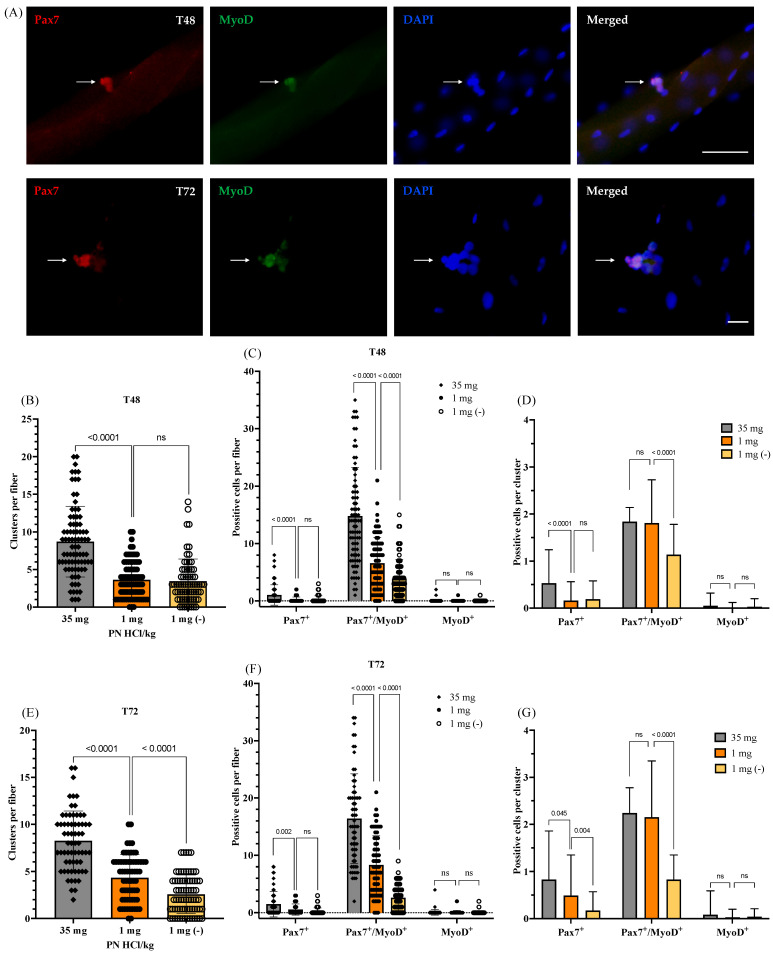
Effects of vitamin B6 deficiency on satellite cell function (proliferation, self-renewal, and differentiation) following 48 and 72 h of culture. (**A**) Representative images of single myofibers after 48 or 72 h of culture co-immunostained for Pax7 and MyoD (arrows) (scale bar = 50 μm (T48) or 20 μm (T72)). (**B**–**D**) Following 48 h of culture, quantified data showed the number of clusters per myofiber (**B**), the number of Pax7^+^, Pax7^+^/MyoD^+^, or MyoD^+^ cells per myofiber (**C**), and the number of the positive cells per cluster recalculated from the (**B**) and (**C**) data (**D**). Four mice were used per group, ≥15 myofibers per mouse; *n* = 83 myofibers (35 mg), *n* = 78 (1 mg), and *n* = 70 myofibers (1 mg (−)). (**E**–**G**) Following 72 h of culture, quantified data showed the number of clusters per myofiber (**E**), the number of Pax7^+^, Pax7^+^/MyoD^+^, or MyoD^+^ cells per myofiber (**F**), and the number of the positive cells per cluster recalculated from the (e) and (f) data (**G**). Four mice were used per group, ≥15 myofibers per mouse; *n* = 65 myofibers (35 mg), *n* = 65 (1 mg), and *n* = 74 myofibers (1 mg (−)). 1 mg (−) indicates the vitamin B6-deficient myofibers cultured in the vitamin B6-free medium. 35 mg PN HCl/ kg and 1 mg PN HCl/kg represent the vitamin B6-supplemented group and the vitamin B6-deficient group, respectively. Values represent the means ± SD. *p* value < 0.05 was considered statistically significant (Unpaired *t*-test). ns = no significance.

To determine the self-renewal, proliferation, and differentiation abilities of satellite cells, we cultured the myofibers for 48 and 72 h and co-immunostained them for Pax7 and MyoD. Incidentally, Pax7-positive/MyoD-negative (Pax7^+^/MyoD^−^) nuclei, Pax7-positive/MyoD-positive (Pax7^+^/MyoD^+^) nuclei, and Pax7-negative/MyoD-positive (Pax7^-^/MyoD^+^) nuclei indicate satellite cells committed towards self-renewal, proliferation, and differentiation, respectively (Figure 3A). During the 48 h culture, the satellite cells divided to form progenies as well as clusters (Figure 3A). As shown in Figure 3B, the number of clusters is fewer in the myofibers of the vitamin B6-deficient mice than in those of the vitamin B6-supplemented mice; however, growing the myofibers in the vitamin B6-deficient medium (1 mg (−)) did not significantly decrease the cluster numbers any further. Notably, the number of satellite cell clusters in each type of myofiber (Figure 3B) is similar to the number of satellite cells observed in them after the 24 h culture (Figure 2C). This suggests that most of the satellite cells on the myofibers isolated from both vitamin B6-deficient and -supplemented mice are insusceptible to early cell death after being activated (24 h) and hence can undergo proliferation towards myogenesis. This result confirms that the vitamin B6-deficient medium does not affect the viability of satellite cells significantly, as observed in case of the 24 h culture study.

As compared to the number of satellite cells observed after 24 h of culture, we did not observe a significant increase in the number of satellite cell clusters after 48 h of culture. However, the number of satellite cell progenies showed a significant increase in myofibers of both vitamin B6-supplemented and -deficient mice; on the contrary, the vitamin B6-deficient medium (1 mg (−)) suppressed the increase in the progeny cells (35 mg: 8.1 ± 4.1 (24 h) vs. 15.7 ± 9.2 (48 h) cells/myofiber, *p* < 0.0001; 1 mg: 3.7 ± 3.1 (24 h) vs. 6.7 ± 4.7 (48 h) cells/myofiber, *p* < 0.0001; 1 mg (−): 3.4 ± 2.2 (24 h) vs. 4.2 ± 3.5 (48 h) cells/myofiber, *p* = 0.16; data not shown). As demonstrated in Figure 3C, upon close observation of each progeny cell type (Pax7^+^, Pax7^+^/MyoD^+^, or MyoD^+^ cells), we found that the number of Pax7^+^/MyoD^+^ cells is lower on the myofibers of the vitamin B6-deficient mice than that of the vitamin B6-supplemented mice. Additionally, the vitamin B6-deficient medium (1 mg (−)) further decreased the number of Pax7^+^/MyoD^+^ cells in the vitamin B6-deficient myofibers (14.8 ± 7.8 (35 mg) vs. 6.6 ± 4.2 (1 mg) cells/myofiber, *p* < 0.0001; 3.9 ± 3.5 (1 mg (−)) cells/myofiber vs. 1 mg, *p* < 0.0001). Thereafter, we calculated the number of Pax7^+^/MyoD^+^ cells per satellite cell cluster (Figure 3D); incidentally, there is no significant difference in this regard between the myofibers of the vitamin B6-deficient and -supplemented mice (1.8 ± 0.3 (35 mg) vs. 1.8 ± 0.9 (1 mg) cells/cluster, *p* = 0.96). On the contrary, the vitamin B6-deficient medium induces a decrease in the Pax7^+^/MyoD^+^ cells per satellite cell cluster in the vitamin B6-deficient myofibers (1.8 ± 0.9 (1 mg) vs. 1.1 ± 0.6 (1 mg (−)) cells/cluster, *p* < 0.0001). These results suggest that vitamin B6 deficiency suppresses the proliferation of satellite cells, but this adverse effect can be reversed if vitamin B6 is available to the cells, for instance in the culture medium as observed in this case.

Subsequently, we observed the effects of vitamin B6 deficiency on the number of Pax7^+^ cells, which indicates the number of satellite cells undergoing self-renewal. As shown in Figure 3C, the number of Pax7^+^ cells is lower in the myofibers of the vitamin B6-deficient mice as compared to that in the myofibers of the vitamin B6-supplemented mice (1.0 ± 1.9 (35 mg) vs. 0.2 ± 0.6 (1 mg) cells/myofiber, *p* < 0.0001). On the contrary, the number of Pax7^+^ cells does not differ significantly between the vitamin B6-deficient myofibers and the myofibers cultured in the vitamin B6-deficient medium (0.2 ± 0.6 (1 mg) vs. 0.3 ± 0.4 (1 mg (−)) cells/myofiber, *p* = 0.41). We calculated the number of Pax7^+^ cells per cluster (Figure 3D) and observed that the myofibers of the vitamin B6-deficient mice exhibited lower numbers of Pax7^+^ cells per cluster as compared to that in the myofibers of vitamin B6-supplemented mice (0.5 ± 0.7 (35 mg) vs. 0.2 ± 0.4 (1 mg) cells/cluster, *p* < 0.0001). Incidentally, the treatment of the myofibers in the vitamin B6-deficient medium did not decrease the number of Pax7^+^ cells per cluster any further than that observed in the myofibers of the vitamin B6-deficient mice (0.2 ± 0.4 (1 mg) vs. 0.2 ± 0.4 (1 mg (−)) cells/cluster, *p* = 0.72). These results suggest that vitamin B6 deficiency suppresses the ability of the satellite cells to undergo self-renewal. This adverse effect is most likely irreversible, as observed after supplementing the culture medium with vitamin B6 in this case.

To determine whether vitamin B6 deficiency affects the differentiation commitment of satellite cells, we observed the number of MyoD^+^ cells in the cultured myofibers. As shown in Figure 3C,D, there are no significant differences in the number of MyoD^+^ cells per myofiber as well as per satellite cell cluster among the different groups of myofibers (35 mg vs. 1 mg vs. 1 mg (−)), thereby suggesting that vitamin B6 deficiency is not likely to affect the ability of satellite cells to commit to differentiation.

Thereafter, we extended the myofiber culture period to 72 h to observe the effects of vitamin B6 deficiency on satellite cell function with progressing time. As shown in Figure 3E, the number of satellite cell clusters in the myofibers of the vitamin B6-deficient mice is lower than that in the myofibers of the vitamin B6-supplemented mice (8.1 ± 3.1 (35 mg) vs. 4.4 ± 2.4 (1 mg) clusters/myofiber, *p* < 0.0001). Additionally, culturing the myofibers in the vitamin B6-deficient medium led to a further significant decrease in the number of satellite cell clusters/myofiber (2.4 ± 2.1 (1 mg (−)) vs. 4.4 ± 2.4 (1 mg) clusters/myofiber, *p* < 0.0001). As compared to the observations after 48 h of culture, the number of satellite cell progenies had slightly increased in the myofibers of both vitamin B6-supplemented and -deficient mice after 72 h of culture, but the vitamin B6-deficient medium suppressed that increase (35 mg: 15.7 ± 9.2 (48 h) vs. 18.0 ± 7.8 (72 h) cells/myofiber, *p* = 0.095; 1 mg: 6.7 ± 4.7 (48 h) vs. 8.9 ± 5.2 (72 h) cells/myofiber, *p* = 0.008; 1 mg (−): 4.2 ± 3.5 (48 h) vs. 2.9 ± 2.4 (72 h) cells/myofiber, *p* = 0.01; data not shown).1 These results suggest that vitamin B6 might play an important role in maintaining the viability of satellite cell progenies during their progression towards myogenesis. As shown in Figure 3F, the number of Pax7^+^/MyoD^+^, Pax7^+^, or MyoD^+^ cells shows a similar trend to that observed after 48 h of culture (Figure 3C). However, as depicted in Figure 3G, the number of Pax7^+^ cells per cluster is the highest in the myofibers of the vitamin B6-supplemented mice and lowest in the vitamin B6-deficient myofibers cultured in the vitamin B6-deficient medium (0.8± 1.0 (35 mg) vs. 0.5 ± 0.9 (1 mg) cells/cluster, *p* = 0.045; 0.2 ± 0.4 (1 mg (−)) cells/cluster vs. 1 mg, *p* = 0.004). These results confirm that the adverse effect of vitamin B6 deficiency on the progression of satellite cells towards self-renewal is irreversible, and the lack of vitamin B6 in the culture medium further worsens this effect. Notably, the vitamin B6-deficient medium (1 mg (−)) induces a further decrease in the number of Pax7^+^/MyoD^+^ cells per cluster after 72 h of culture (Figure 3G), as compared to that after 48 h of culture (Figure 3D). In contrast, the number of Pax7^+^/MyoD^+^ cells per cluster in the myofibers of the vitamin B6-deficient or -supplemented mice cultured in the normal medium remained unchanged or was slightly increased. This result indicates that the vitamin B6-deficient medium further decreases the number of satellite cell clusters per myofiber, which is consistent with the result shown in Figure 3E.

In conclusion, it seems likely that vitamin B6 deficiency suppresses the proliferation and self-renewal of the satellite cells during myogenesis, and with the progression of time, the vitamin B6 deficiency is likely to further impact the viability of satellite cell progenies.

### 3.3. Effects of Vitamin B6 Deficiency on Amino Acid and Peptide Metabolisms in Muscles

#### 3.3.1. Changes in Amino Acids and Imidazole Dipeptides in Skeletal Muscles

We attempted to explain the pathways that lead to vitamin B6 deficiency-induced impairment of the number and function of satellite cells. Since vitamin B6 is predominantly stored in muscles, and it plays an important role in amino acid metabolism, we investigated the impact of vitamin B6 deficiency on amino acid composition in the skeletal muscles. We observed that the levels of half of the proteinogenic amino acids, namely alanine, arginine, histidine, isoleucine, leucine, lysine, methionine, phenylalanine, serine, tyrosine, and valine, are significantly decreased in the vitamin B6-deficient mouse muscles (Table 2). Additionally, the levels of taurine, which is a non-proteinogenic amino acid naturally present in high amounts in the skeletal muscles, are significantly lower in the vitamin B6-deficient mouse muscles as compared to that in the vitamin B6-supplemented mouse muscles.

**Figure 4:**
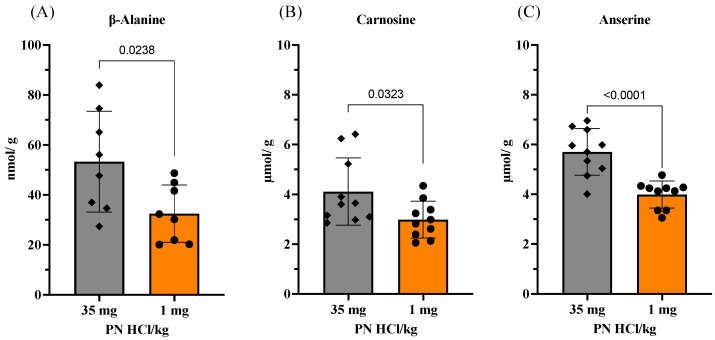
Effects of vitamin B6 deficiency on levels of imidazole dipeptides and their substrate, β-alanine, in skeletal muscles. (**A**) Levels of β-alanine, (**B**) levels of carnosine, and (**C**) levels of anserine. 35 mg PN HCl/ kg and 1 mg PN HCl/kg represent the vitamin B6-supplemented group and the vitamin B6-deficient group, respectively. Values represent the means ± SD (*n* = 10). *p* value < 0.05 was considered statistically significant (unpaired *t*-test).

This change in the taurine levels led us to further explore whether vitamin B6 deficiency had any effect on β-alanine, which is another predominant non-proteinogenic amino acid in skeletal muscles, and its imidazole dipeptide levels. As shown in Figure 4A–C, the levels of β-alanine and its imidazole dipeptides, namely carnosine (β-alanyl-L-histidine) and anserine (β-alanyl-N1-methyl-L-histidine), are significantly lower in the vitamin B6-deficient mouse muscles as compared to that in the vitamin B6-supplemented mouse muscles. These observations are in good agreement with our previous study that reported that the vitamin B6-deficient diet induces a greater decrease in the levels of proteinogenic amino acids as well as β-alanine, carnosine, and anserine in rat heart muscles as compared to the changes induced by the vitamin B6-supplemented diet [17].

#### 3.3.2. Regulation of Carnosine Synthesis in C2C12 Muscle Cells by Vitamin B6

**Figure 5:**
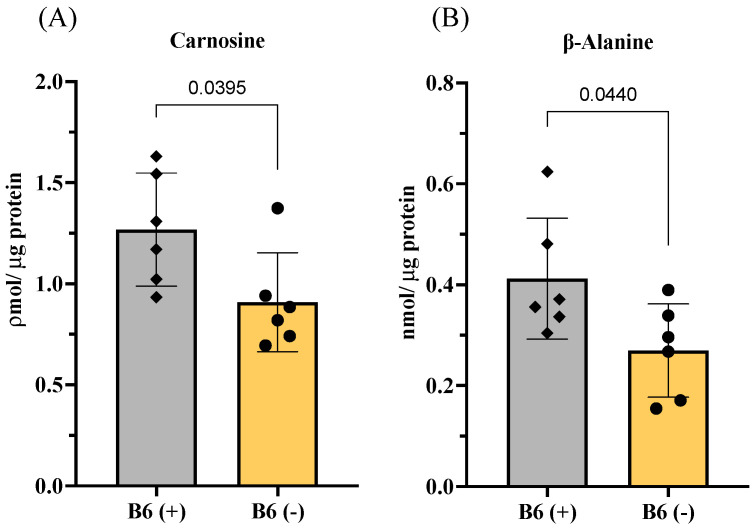
Effects of vitamin B6 on carnosine biosynthesis in C2C12 muscle cells. (**A**) Levels of carnosine and (**B**) levels of β-alanine. B6 (+) and B6 (−) represent the normal medium with vitamin B6 (PN) or the vitamin B6 (PN)-deficient medium, respectively, used in C2C12 cell culture. Values represent the means ± SD (*n* = 6 wells from a 6-well plate). *p* value < 0.05 was considered statistically significant (Unpaired *t*-test).

Since carnosine is the most well-known and essential muscle peptide, we further sought to determine whether vitamin B6 directly regulates the biosynthesis of this peptide in muscle cells. Using C2C12 muscle cells, we observed that vitamin B6-deficient medium (B6 (-)) significantly decreases the levels of carnosine and its substrate, β-alanine, in the muscle cells (Figure 5A,B).

In conclusion, our observations indicate that vitamin B6 deficiency impairs amino acid metabolism as well as muscle peptide synthesis.

## 4. Discussion

Poor nutrition is known to play a role in the development of sarcopenia. Until now, the nutrients that have been studied most extensively for their roles in improving or preventing sarcopenia are proteins and amino acids, followed by vitamin D and omega 3 fatty acids [3,5,6]. Interestingly, among the 13 vitamins for humans, vitamin D is the only one that has been extensively studied at both physiological and cellular levels. However, recent research suggests that other nutrients, such as antioxidants, manganese, vitamin A, vitamin E, and vitamin B6, also play a role in the prevention or improvement of sarcopenia [4,7,8,9,10,11,12,13]. Recent human studies (2016–2021) in European countries as well as Japan have demonstrated that the low intake and low levels of vitamin B6 coexist with sarcopenia [7,8,9,10,11]. In 2021, Grootswagers et al. [8] reported that increased vitamin B6 intake in older European people is related to improvements in the chair rest test, thereby reflecting muscle power, as well as handgrip strength, which reflects muscle strength. In a 2020 study, over 50% of the older German subjects were found to have a low dietary intake of vitamin B6, and approximately 40% of them had low blood vitamin B6 levels [10]. In a 2016 study, it was reported that older, sarcopenic Dutch people have a low intake of vitamin B6 [11]. Similarly, in 2020, older Japanese people with a low skeletal muscle index, which reflects muscle loss, were also found to have a low vitamin B6 intake [9]. It has been hypothesized that the low vitamin B6 intake as well as its low levels in older or sarcopenic people is possibly due to the low intake of proteins, one of the main food sources for vitamin B6, in the elderly [10]. Additionally, this low intake or status of vitamin B6 in the elderly may increase oxidative stress that is reflected by high homocysteine levels and subsequent muscle protein degradation in the old, sarcopenic people, thereby leading to a decline in muscle strength and physical function [11]. Although these recent studies shed light on the potential role of vitamin B6 in sarcopenia, there is not enough literature in this regard, and further studies are necessary at both physiological and cellular levels. Therefore, in this study, we examined the effects of vitamin B6 on muscle stem cells i.e., the satellite cells that play an important role in skeletal muscle regeneration.

**Figure 6:**
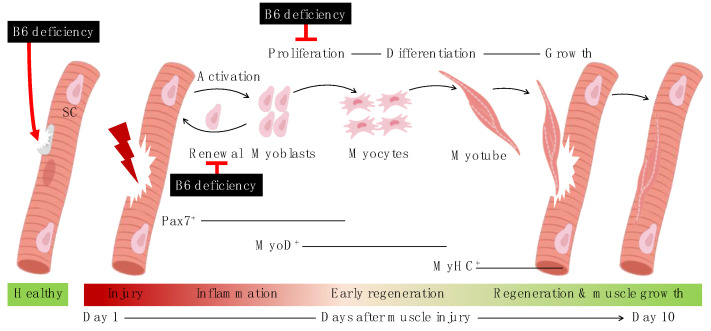
Events during myogenic progression upon injury and effects of vitamin B6 deficiency on satellite cells. SC, satellite cells; B6, vitamin B6 (PN HCl). Created with BioRender.com (accessed on 12 December 2021).

In response to injury or aging, skeletal muscle undergoes regeneration to repair itself. Its regenerative capacity is owing to a resident stem cell population, namely the satellite cells that are located on the muscle fibers or myofibers [23]. Under normal conditions, the satellite cells are in a quiescent or inactive state, but in response to an injury or any pathological condition, they get activated and undergo self-renewal, which helps to return the satellite cells to their quiescent state to replenish the satellite cell pool, and induce their proliferation and differentiation to fuse the damaged myofibers and produce new ones for muscle regeneration [23], as shown in Figure 6. Although the role of satellite cells in muscle mass maintenance during aging is debatable, the concept of age-related decrease in the muscle repair efficiency and muscle regeneration capacity due to a decrease in the number of quiescent satellite cells, thereby contributing to the development of sarcopenia, is still widely accepted [6,23].

In the present study, we observed that vitamin B6 deficiency significantly decreased the number of quiescent satellite cells on myofibers (Figure 2B). This observation may explain the association between low vitamin B6 intake or its low level in the body and sarcopenia, as observed in certain previous human studies [7,8,9,10,11]. This decrease in the number of quiescent satellite cells due to vitamin B6 deficiency may be partly attributed to the impaired satellite cell synthesis or a defect in satellite cell proliferation capacity. The mice used in this study were in the adolescent stage (5–13 w old), wherein rapid growth occurs for the whole body development. For this, protein synthesis is highly required all over the body, including the muscle and satellite cells. Vitamin B6 plays a crucial role in protein synthesis because it can regulate amino acid metabolism [14]. As shown in Table 1 and Table 2, vitamin B6 deficiency-induced depletion of PLP and amino acid pools might impair protein synthesis that is essentially required for satellite cell production. Furthermore, a recent study demonstrated that in mice at 4–5 weeks of age, their satellite cells are not fully quiescent and >60% of the satellite cells are still in a myogenic state (Pax7^+^/MyoD^+^) [24]. Thus, feeding the vitamin B6-deficient diet to mice at 5 weeks of age in our current study might affect satellite cell proliferative or myogenic capacity, consequently reducing the satellite cell pool observed at 13 weeks of age (Figure 2).

In addition to the main function of being a cofactor for enzymes in amino acid metabolism, vitamin B6 also acts as an antioxidant to efficiently protect the cells against increasing reactive oxygen species, which cause oxidative stress [14,25]. Moreover, vitamin B6 is a modulator for maintaining oxidative balance by regulating the homeostasis of other antioxidants, such as carnosine, anserine (Figure 4) [17,26], glutathione [27], and hydrogen sulfide [25,28], and by preventing the increase in homocysteine, a factor causing oxidative stress [11,29]. Furthermore, vitamin B6 deficiency downregulates the expressions of antioxidant genes, such as the ones encoding heme oxygenase-1 (*HO-1*), superoxide dismutase 2 (*SOD2*), glutathione peroxidase 1 (*GPX1*), and glutathione S-transferase (*GST*), as well as their master transcription factor, nuclear factor erythroid 2-related factor 2 (Nrf2), in rodents [30]. Although quiescent satellite cells have well-developed protective mechanisms that confer resistance against toxic substances or a detrimental environment, such as oxidative stress [31,32], vitamin B6 deficiency might induce extrinsic, environmental changes that are harmful to the quiescent satellite cells or intrinsic changes within the satellite cells that make them vulnerable to the harmful environmental conditions, such as oxidative stress. As demonstrated, the abnormal accumulation of some nutrients, such as iron, which induces oxidative stress in skeletal muscle, leads to a decline in the quiescent satellite cell population [33]. With respect to energy metabolism, quiescent satellite cells predominantly derive their energy requirements from glycolysis [34], where vitamin B6 (PLP) is a cofactor for glycogen phosphorylase that is responsible for glycogen breakdown [34]. Therefore, vitamin B6 deficiency might decrease the accessibility of quiescent satellite cells to their main energy source, thereby decreasing the proliferation and integrity of satellite cells. However, further studies are necessary to provide direct evidence determining if vitamin B6 deficiency induces oxidative stress environment deleterious to satellite cells subsequent to a decline in the cell pool. Further studies on the vitamin B6-deficient feeding on mice and a global histological analysis of skeletal muscles at each time point between 5 and 13 weeks of age will warrant the direct evidence confirming the adverse effects of vitamin B6 deficiency on satellite cell proliferative or myogenic capacity, leading to a decline in satellite cell pool.

With respect to self-renewal, we observed that vitamin B6 deficiency suppresses this ability in the satellite cells. Additionally, this adverse effect is most likely irreversible because the addition of vitamin B6 in the culture medium could not help to overcome the self-renewal defect. Hence, it can be hypothesized that vitamin B6 deficiency possibly affects the intrinsic factors responsible for the self-renewing ability of satellite cells. Moreover, we found that the lack of vitamin B6 in the culture medium further worsened the self-renewal capability of the satellite cells. This suggests that vitamin B6 deficiency during myogenesis might downregulate Pax7 expression or decrease its transcriptional activity because high expression of Pax7 and downregulation of the expression of myogenic regulatory factors, such as Myf5 and MyoD, are responsible for inducing the preferential self-renewal of satellite cells [34]. It has been previously reported that the acetylation of Pax7 regulates the self-renewal ability of the satellite cells; incidentally, Pax7 acetylation is regulated by the effector, acetyl-CoA, and the suppressor, NAD^+^ [35]. This information led us to hypothesize that vitamin B6 deficiency might modulate the availability of acetyl-CoA and NAD^+^, since both of which are mediators of the energy metabolism pathways in which vitamin B6 is an important cofactor for the enzyme hydrolyzing glycogen. This hypothesis may be true with respect to the decrease in the quiescent satellite cell numbers in vivo, but it cannot explain the observations in the case of ex vivo myofiber culture. Regardless of the presence or absence of vitamin B6 in the culture medium, glucose is always available; hence, myofibers should be able to produce acetyl-CoA via glycolysis. However, we cannot neglect the possibility that vitamin B6 deficiency may decrease mitochondrial functioning, thereby leading to a defect in the energy metabolism pathway, which, in turn, may affect the homeostasis of acetyl-CoA and NAD^+^. In fact, previous studies have demonstrated that vitamin B6 deficiency downregulates the gene expression of heat shock protein 60 [8], which is a mitochondrial molecular chaperone essential for maintaining proper mitochondrial function, including energy production [36]. Incidentally, fibroblast growth factor receptor (FGFR) and p38α/p38β mitogen-activated protein kinase (p38α/β MAPK) signaling are essential for the self-renewal of satellite cells. It has been reported that impaired response of FGFR and hyper-elevated p38α/β MAPK activity induce cell-intrinsic defects that affect the self-renewal of satellite cells isolated from aged mice, and these defects cannot be reversed by exposing these satellite cells to a young environment [37]. Another study reported that fibroblast growth factor 2 (FGF2), which is one of the FGFR ligands, was upregulated in myofibers of aged mice [38], and this could be a compensatory mechanism for the blunt response of FGFR observed in the aged mice [37]. In fact, the increased abundance of the myofiber-derived FGF2 in aged muscles led to a loss of the self-renewing capacity of the aged satellite cells [38]. Additionally, the FGF2 mRNA level was significantly increased in newborn pup from rat dams fed on a vitamin B6-deficient diet [38]. This increased FGF2 expression is most likely a compensatory response to the vitamin B6 deficiency, so that the newborn pups can achieve a normal growth; this is consistent with the fact that vitamin B6 is vital for adolescent growth [18,37]. Therefore, with respect to the observations of the current study, it may be suggested that GF2 might be upregulated in the vitamin B6-deficient myofibers subsequent to a disturbance in FGFR- p38α/β MAPK signaling; this may have caused the cell-autonomous loss of satellite cell self-renewal that could not be restored even after exposing the myofibers to an optimal vitamin B6-enriched environment. Hence, further investigations are necessary to understand the association between vitamin B6 deficiency and the FGFR- p38α/β MAPK signaling pathway with respect to the self-renewal of satellite cells.

In this study, we also observed that vitamin B6 deficiency decreases the proliferative capacity of satellite cells; however, this adverse effect could be reversed by exposure to a vitamin B6-enriched environment (Figure 3D,G (Pax7^+^/MyoD^+^)). We hypothesized that these proliferative defects induced by the vitamin B6 deficiency might be due to an imbalance in the oxidative defense mechanism of the cells. In a recent study including the metabolomics analysis of primary human skeletal muscle cells, it was reported that among the 17 metabolic pathways that were significantly altered during myogenic progression, vitamin B6 and glutathione metabolisms were highly upregulated [39]. In fact, during myogenic progression, there was an increased usage of pyridoxal, a precursor of PLP (the active form of vitamin B6), which possibly indicated an increased need for PLP, and an increased production of oxidized glutathione, which possibly reflected a high rate of oxidative activity [39]. Combined with our present study, these observations indicate that a homeostasis of vitamin B6 metabolism and oxidative defense system is essential to ensure normal myogenesis in the satellite cells. Future studies demonstrating directly effects of vitamin B6 deficiency on satellite cell proliferative capacity through oxidative stress pathways are needed to warrant the hypothesis.

Another possible cause for the reduced proliferation of the satellite cells in vitamin B6-deficient myofibers may be the high responsiveness of the vitamin B6-deficient satellite cells to glucocorticoid receptor (GR)-mediated signaling pathways. Incidentally, PLP acts as a transcriptional modulator by directly interacting with GR and thereby suppressing the binding between GR and nuclear factor 1. Subsequently, the transcription of glucocorticoid-regulated target genes gets suppressed; as demonstrated that the cells cultured in vitamin B6-deficient state exhibited higher responsiveness to a GR ligand, dexamethasone [40]. Glucocorticoid drugs, such as dexamethasone, can induce muscle atrophy, suppress the proliferation and differentiation of myoblasts, and reduce myogenic gene expression [41]. Dexamethasone treatment was found to decrease the proliferation of satellite cells through upregulation of myostatin expression, which, in turn, downregulated some myogenic regulatory genes [42]. In fact, after 3–4 d of culturing the myofibers on dexamethasone, there was a decrease in the number of proliferating C2C12 cells, thereby indicating cell death or decreased cell viability [43]. This result is consistent with our observation that the number of proliferating satellite cells on the myofibers cultured in the vitamin B6-deficient medium was significantly lower at 72 h after culture than that observed at 48 h (Figure 3C,D,F,G). Hence, it can be postulated that vitamin B6 deficiency might enhance the transcriptional responses to glucocorticoids in satellite cells, which may increase the expression of genes involved in the downregulation of satellite cell proliferation. Since some nutrients, such as vitamin D, have been found to prevent muscle atrophy induced by dexamethasone [44], it will be interesting to determine whether vitamin B6 deficiency affects glucocorticoid-induced muscle atrophy.

The decrease in the levels of various amino acids in the vitamin B6-deficient muscles, especially the branched-chain amino acids, may also contribute to the defects in the satellite cell function. For instance, leucine significantly promotes the proliferation and differentiation of satellite cells partly through the mammalian target of rapamycin complex 1 (mTORC1) pathway [44]. Lysine deficiency was found to induce apoptosis in the satellite cells isolated from the muscles of piglets, and lysine supplementation promoted satellite cell proliferation via the mTORC1 pathway [45,46]. Moreover, a recent study demonstrated that taurine supplementation can increase the viability of C2C12 cells that have been exposed to reactive oxygen species (ROS) as well as promote their proliferation and differentiation [47]. Branched-chain amino acid aminotransferase, an enzyme involved in the catabolism of branched-chain amino acids, is essential for maintaining the self-renewal of mouse embryonic stem cells [48]. In the current study, along with the changes in the amino acids, muscle peptides carnosine and anserine were also downregulated in the vitamin B6-deficient myofibers (Figure 4); in fact, their biosynthesis was confirmed to be directly dependent on vitamin B6 in the C2C12 cells (Figure 5). Carnosine and anserine are naturally occurring dipeptides present in high concentrations in the mammalian skeletal muscle [49]. Their multifunctional roles in skeletal muscles have been well addressed through intracellular pH buffering, moderating the energy metabolism, intracellular Ca^2+^ level regulation, and antioxidant activity [49]. Although there is limited information regarding their roles in muscle satellite cells, there is some evidence suggesting their potential role in the proliferation of human stem cells and the prevention of senescence in human skeletal myoblasts [50,51,52]. A recent study demonstrated that vitamin B6 and carnosine metabolisms were highly upregulated during myogenic progression of primary human skeletal muscle cells [39]. Transcriptome analysis of human skin fibroblasts has indicated that carnosine stimulates cell proliferation by modulating the expression of genes involved in the various stages of cell cycle, including initiation of DNA replication, chromosome condensation during prometaphase, initiation of mitosis, and spindle formation [53]. In conclusion, it may be suggested that the overall decrease in amino acids as well as dipeptides, carnosine and anserine, contributes to the defects in satellite cell function as observed in vitamin B6-deficient mice of the present study. Further investigations are needed to test this assumption.

The limitations of this study are as follows: (1) the vitamin B6-recommended level (7 mg PN HCl/kg diet) was not included in the diet, and (2) the culture medium was not changed daily, which possibly limited the availability of nutrients for the satellite cells during myogenic progression. In addition, recent studies [54,55] have suggested that MyoD may not be a suitable indicator for an accurate readout of satellite cell self-renewal and differentiation. Thus, in the future, instead of MyoD, myogenin should be used with Pax7 to warrant the proper analysis of self-renewing versus differentiated satellite cells; besides, quantifying Ki67 or BrdU expression with Pax7 will warrant an accurate analysis of proliferating satellite cells instead of using only Pax7^+^/MyoD^+^. Hence, further studies are required to address these limitations and test all the hypotheses raised in this study regarding the effects of vitamin B6 deficiency on the number and function of the satellite cells. In fact, future studies involving transcriptomic and metabolomic analyses are essential to elucidate the mechanisms of vitamin B6 underlying the regulation of satellite cell function. Further studies on the role of vitamin B6 in muscle regeneration and muscle atrophy will warrant the application of this vitamin in nutritional interventions for sarcopenia treatment in the future.

## 5. Conclusions

This study demonstrates that vitamin B6 deficiency induces a number of changes in the satellite cells associated with myofibers, including a decline in the quiescent satellite cell pool, an irreversible defect in their self-renewal, and a reversible defect in the proliferation of activated satellite cells. Moreover, vitamin B6 deficiency stimulates a decrease in the levels of amino acids and peptides, namely carnosine and anserine, in the muscles, which, in turn, may exert adverse effects on satellite cell viability and function. Furthermore, we demonstrated that carnosine biosynthesis in muscle cells is dependent on the availability of vitamin B6. In fact, our study provides the first evidence for the role of vitamin B6 in satellite cell function and its potential application in the prevention of sarcopenia.

## Figures and Tables

**Table 1:** Food intake, body weight, muscle mass, and PLP levels.

Groups (PN HCl/kg)	B6-Supplemented (35 mg)	B6-Deficient (1 mg)
Body weight		
Initial (g)	31.5 ± 2.8	31.5 ± 2.2
Final (g)	55.3 ± 5.5	52.3 ± 5.7
Food intake (g/day/mouse)	4.1 ± 0.2	3.9 ± 0.3
Muscle weight		
Gastrocnemius (mg)	206.9 ± 23.6	205.6 ± 24.6
Selous (mg)	14.4 ± 16.7	10.0 ± 2.9
PLP levels		
Plasma (nmol/mL)	3.03 ± 1.09	0.98 ± 0.21 ^0.003^
Gastrocnemius muscle (nmol/g)	9.9 ± 1.6	4.5 ± 0.6 ^<0.0001^

Values are expressed as the means ± SD (*n* = 10). *p* value < 0.05 was considered statistically significant (unpaired *t*-test). B6, vitamin B6; PN, pyridoxine; PLP, pyridoxal 5′-phosphate.

**Table 2:** Significantly changed amino acids in skeletal muscles in response to dietary vitamin B6 levels.

Amino Acids	Concentrations (nmol/g)		*p* Values
	B6-Supplemented (35 mg)	B6-Deficient (1 mg)	
Alanine	1469± 559	735 ± 118	0.021
Arginine	85 ± 18	38 ± 15	0.002
Histidine	99 ± 40	51 ± 6	0.029
Isoleucine	97 ± 39	43 ± 8	0.016
Leucine	153 ± 62	70 ± 11	0.018
Lysine	178 ± 34	110 ± 35	0.015
Methionine	77 ± 31	38 ± 9	0.025
Phenylalanine	76 ± 30	34 ± 6	0.016
Serine	296 ± 114	144 ± 31	0.021
Tyrosine	90 ± 33	46 ± 9	0.020
Valines	147 ± 65	74 ± 9	0.037
Taurine	22,294 ± 7218	12,347 ± 789	0.016

Values are expressed as the means ± SD (*n* = 5). *p* value < 0.05 was considered statistically significant (unpaired *t*-test). B6, vitamin B6; 35 mg, 35 mg PN HCl/kg; 1 mg, 1 mg PN HCl/kg; PN, pyridoxine.

## Data Availability

The datasets used and/or analyzed during the current study are available from the corresponding author on reasonable request.

## Supplementary Materials

The following are available online at https://www.mdpi.com/article/10.3390/nu13124531/s1, Figure S1: Morphology of vitamin B6-supplement and -deficient gastrocnemius (GAS) muscle cross sections.
