# Supplementary material for: Satellite Cells Exhibit Decreased Numbers and Impaired Functions on Single Myofibers Isolated from Vitamin B6-Deficient Mice
Source: Nutrients. 2021 Dec 17;13(12):4531. doi: 10.3390/nu13124531 (PMC8705767; doi:10.3390/nu13124531)
Supplement: Supplementary file 1 [file nutrients-13-04531-s001.zip › nutrients-1482457-supplementary.pdf]

Supplementary Material

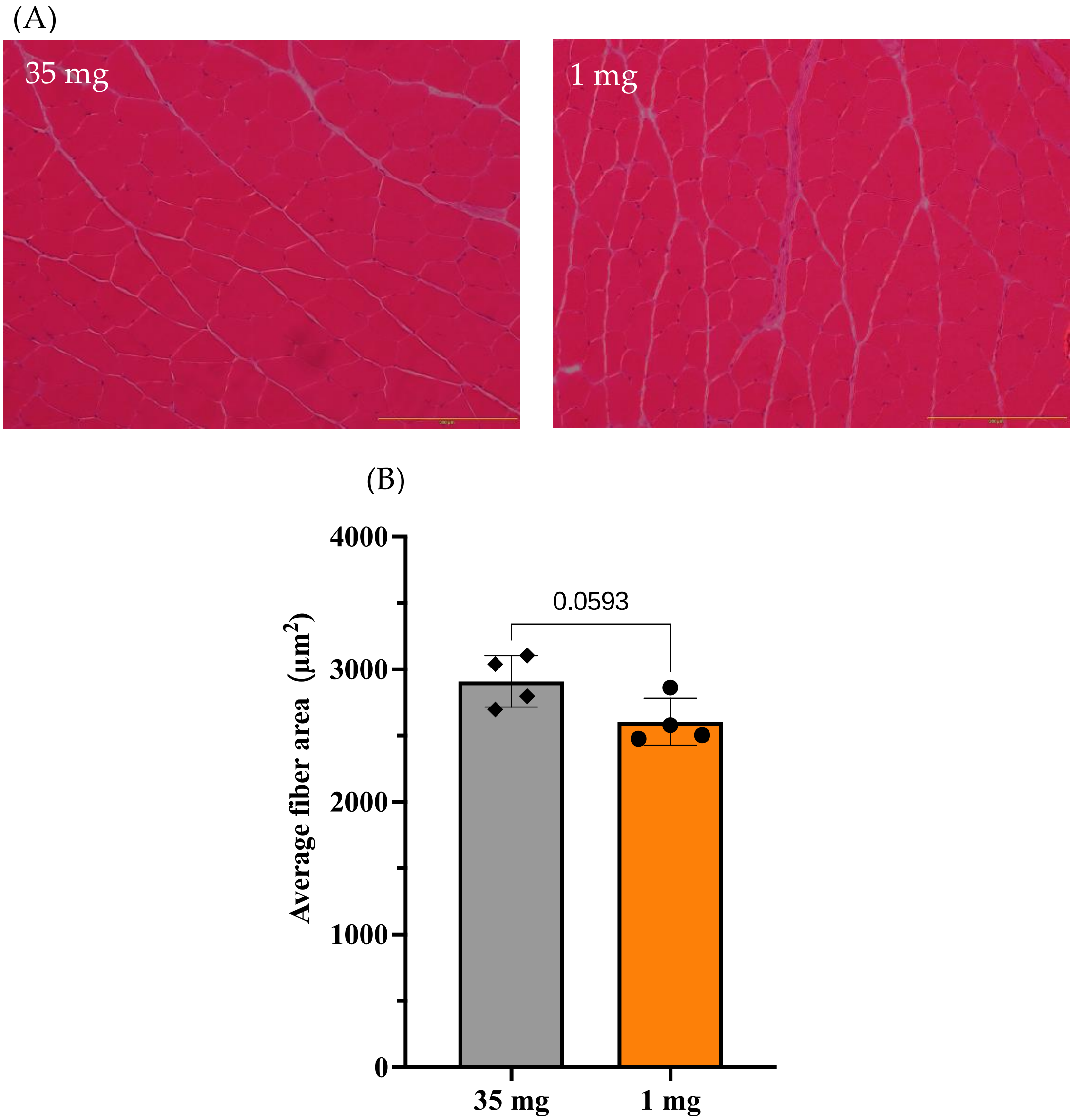

Figure S1. Morphology of vitamin B6-supplement and -deficient gastrocnemius (GAS) muscle cross sections. (A) Representative images depicting fiber size of myofibers from hematoxylin and eosin (H&E) stain. Scale bar 200 μm. (B) The myofiber area of muscles from mice fed the vitamin B6-deficient diet was slightly smaller ($P = 0.0593$) than that from mice fed the vitamin B6-supplement diet. For H&E stains, standard staining protocols were used, and the stains were completed on GAS muscle sections. An average of 200 fibers were examined per muscle section ($n = 4$ mice/group). Values represent the means ± SD. P value < 0.05 was considered statistically significant (unpaired t-test).
